# A Systematic Review and Meta-Analysis of Harmonic Scalpel Versus Conventional Techniques of Appendiceal Stump Closure in Laparoscopic Appendicectomy

**DOI:** 10.7759/cureus.28759

**Published:** 2022-09-03

**Authors:** Nitinkumar Borkar, Charu Sharma, Debajyoti Mohanty, Subrata K Singha

**Affiliations:** 1 Paediatric Surgery, All India Institute of Medical Sciences, Raipur, Raipur, IND; 2 General Surgery, All India Institute of Medical Sciences, Raipur, Raipur, IND; 3 Anaesthesiology, All India Institute of Medical Sciences, Raipur, Raipur, IND

**Keywords:** meta-analysis, harmonic scalpel, appendicectomy, sutureless, laparoscopic

## Abstract

Acute appendicitis is one of the most commonly encountered surgical emergencies worldwide. The laparoscopic approach for managing acute appendicitis is gaining popularity over open appendicectomy in the current surgical practice. The advantages of laparoscopic appendectomy are early recovery, fewer wound complications, less pain and better cosmesis. One of the most critical steps in laparoscopic appendicectomy is a secure appendicular stump closure. Life-threatening postoperative complications are often encountered following the breakdown of appendicular stump closure. There are several methods to achieve appendicular stump closure such as intra-corporeal knotting, endoloops, external corporeal knotting and pushing knot inside, endoscopic linear cutting stapler (endo GIA), and endoclips. A meta-analysis on the technique of appendicular stump closure in laparoscopic appendicectomy failed to demonstrate the superiority of one method over the other. In the last few years, many authors have evaluated the outcome of sutureless appendicectomy performed using devices like a harmonic scalpel. This systematic review and meta-analysis is aimed to summarise the current evidence regarding the utility and safety of harmonic scalpel in sutureless appendicectomy.

This systematic review and meta-analysis was conducted as per the preferred reporting items for systematic review and meta-analyses (PRISMA) guidelines. A systematic, detailed search was carried out by the authors in the electronic database, including Medline, Embase, CENTRAL, Scopus, Google scholar and clinical trial registry. Studies were selected and compared based on outcomes such as operative time, hospital stay, postoperative paralytic ileus, wound infection, and total complications. Statistical analysis was performed using the random effect model, fixed-effect model, pooled risk ratio, pooled mean difference and I^2^ heterogeneity.

Four comparative studies with a total of 642 patients (376 male and 266 females) were included in the analysis. There were 359 patients in the conventional technique of appendicular stump closure group and 283 patients in the harmonic scalpel for appendicular stump closure group. Pooled analysis of the outcome measure of total complications showed that the use of harmonic scalpel for closure of appendiceal stump does not result in an increased incidence of complications as compared to the conventional technology of appendiceal stump closure. Pooled analysis of the outcome measure of mean operative time revealed a statistically significant reduction in the operative time in the patients where harmonic scalpel has been used for the management of appendiceal stump as compared to conventional methods (pooled mean difference of -12.96 with 95% CI -15.42, -10.50). Appendiceal stump closure during laparoscopic appendectomy by harmonic scalpel (HS) is comparable with the conventional techniques in terms of hospital stay, wound infection, postoperative paralytic ileus, and total complications. The use of a harmonic scalpel for closure of appendicular stump is associated with a reduction of the mean operative time of laparoscopic appendicectomy.

## Introduction and background

Acute appendicitis is one of the most commonly encountered surgical emergencies worldwide. The peak incidence is seen in the second and third decades of life. The lifetime risk of acute appendicitis is 6.7% in females and 8.6% in men [1]. The laparoscopic approach has replaced open appendicectomy for managing acute appendicitis in current surgical practice. The advantages of laparoscopic appendectomy (LA) are early recovery, fewer wound complications, less pain and better cosmesis [2]. One of the most critical steps in LA is a secure appendicular stump closure. There can be life-threatening postoperative complications following suboptimal closure of the appendicular stump. Hence, the proper closure of the appendicular stump is essential for a successful LA. There are several methods to achieve this objective such as intra-corporeal knotting, endoloops, external corporeal knotting and pushing knot inside, endoscopic linear cutting stapler (endo GIA), and endoclips. A recent meta-analysis on the method of appendicular stump closure in LA failed to demonstrate the superiority of one technique over the other [3]. The use of endoloops and polymer clips for stump closure is common due to the ease of availability and affordable pricing while the use of Endo GIA is limited due to the high cost and the need for an additional 12 mm port for introducing the Endo GIA device. The foreign materials used for securing the appendicular stump can induce intense inflammation inside the abdominal cavity leading to the development of adhesive intestinal obstruction [4,5].

In the past few years, many authors have evaluated sutureless appendicectomy using devices like a harmonic scalpel (HS) and bipolar coagulation [6-8]. The advantage of this technique is the elimination of foreign material-related postoperative complications. The HS is an ultrasonic energy-powered instrument used in both open and laparoscopic procedures for tissue cutting and coagulation. It is a versatile instrument that performs dissection, cutting and sealing with a single-hand instrument. Its use is associated with limited thermal spread, lesser tissue charring and minimal smoke formation compared to traditional electrosurgical instruments. Several reports demonstrate that HS can be a safe and handy instrument for sealing and resecting luminal structures such as the appendix and cystic duct [6,7,9]. The base of the appendix is reported to be effectively sealed with an energy device like bipolar or ultrasonic coagulator set at lower power and in a staggered manner [7,8]. Before its introduction to clinical application, the safety and efficacy of this technique were demonstrated in rats by Aslan et al. [10] who reported that coagulation of the appendix stump with a bipolar energy device did not allow any leakage of intraluminal contents. An ex vivo study by Yavuz et al. [11] evaluated the appendix stump opening pressure in the right colectomy and subtotal colectomy specimens. Following appendectomy, the stump closure was performed with either silk ligature or energy devices like LigaSure and HS. They concluded that performing appendectomies using LigaSure and HS can be as effective as the conventional methods. Amidst the promising results of various studies favouring HS for sutureless appendectomy, Gozeneli et al. [12] reported incomplete appendix stump closure with ultrasonic instruments in their ex vivo study of 20 patients. The consensus report on using HS for sutureless appendicectomy is far from convincing. This systematic review and meta-analysis is aimed to summarise the current evidence regarding the utility and safety of a harmonic scalpel in sutureless appendicectomy.

## Review

Materials and methods

Main Objective

To assess the safety and efficacy of harmonic scalpel in sutureless appendicectomy compared to the conventional techniques of securing the base of the appendicular stump in laparoscopic appendicectomy.

Search Process

The present systematic review and meta-analysis was performed using the PRISMA (preferred reporting items for systematic review and meta-analysis) guidelines [13]. This review was not previously registered in any prospective register and database. Preliminary searches were performed by two authors (CS, NB) to confirm the absence of any similar systematic review on the topic.

Electronic Searches

Two authors (DM, NB) conducted a comprehensive literature search to identify all published and unpublished randomized controlled trials (RCT) and comparative studies. The search was run separately in the following databases: Cochrane Central Register of Controlled Trials (CENTRAL) in The Cochrane Library, MEDLINE, Embase, and Scopus till 31 July 2022.

We searched the WHO International Clinical Trials Registry Platform (ICTRP) and the US National Institutes of Health Ongoing Trials Register (clinicaltrials.gov) for completed and ongoing studies. To complete the search for relevant studies, we scanned the reference lists of all full-text papers and used judgment to decide whether to pursue these further to include in our list [14]. We also performed a forward citation search of the included articles using Google Scholar. The search keywords included- Harmonic scalpel OR Sutureless OR Loop Knots OR Endoloops OR Ultrasonically activated scalpel OR Clipless OR Extra corporeal knotting AND Laparoscopic Appendicectomy OR Laparoscopic Appendectomy. Once screened, duplicate entries were removed and the remaining articles were screened for eligibility to be included in the review. Searches were rerun before the final analysis.

Eligibility criteria

The inclusion criteria are given in Table 1.

**Table 1:** Inclusion criteria in PICO format HS: Harmonic scalpel

PICO format	Criteria
Participants (P)	Patients with appendicitis who had undergone laparoscopic appendicectomy
Intervention (I)	Use of HS for sutureless appendicectomy
Comparison (C)	Conventional techniques (CT) of securing the base of the appendicular stump
Outcome (O)	Operative time, duration of hospital stay, postoperative paralytic ileus, wound infection/post site infection, and total complications.

Studies using HS for only mesoappendix ligation without stump closure, sutureless appendicectomy using diathermy energy, and thermal fusion technique were excluded from this review. We included only RCTs and comparative studies for this review. Studies with a single-arm trial, case series, expert opinion, letters to editors, and studies where no outcome of interest was reported were excluded from our meta-analysis.

Data collection and analysis

Selection of Studies

Two review authors (NB, CS) independently screen the titles and abstracts of the included studies. All the potentially eligible studies were retrieved and assessed for the full texts. Discrepancies regarding the inclusion of any study for the review were resolved by discussion with the senior author (DM).

Data Extraction and Management

Two review authors (NB, DM) independently extracted and entered data onto an electronic data collection form and another author (CS) checked the data for accuracy. Later, data were entered into Review Manager 5.4 (RevMan 2020) by another author (NB). From each included study, we collected information on setting, period of study, number of participants within each arm, mean/median age, number of participants of different gender, duration of surgery, length of hospital stay, and days of paralytic ileus, wound infection, and number of reoperations. We used RevMan 5.4 to perform all data analysis.

Methodological Quality Assessment

Two independent reviewers (SS and DM) conducted the methodological quality assessment utilizing the modified Downs and Black scale [15]. This checklist can evaluate both RCTs and non-controlled trials. The scale has 27 points of assessment yielding a total score of 0-28. Downs and Black score ranges were given for corresponding quality levels as previously reported: excellent (26-28); good (20-25); fair (15-19); and poor (<14). The reviewers' results were compared by a third reviewer (NB) and discrepancies were resolved in a consensus meeting. Followed by this, the kappa statistics were used to adjudge the interobserver reliability.

Measures of Treatment Effect

We analysed the data using Review Manager (RevMan) [Computer program], Version 5.4, The Cochrane Collaboration, 2020. Dichotomous outcomes were expressed as risk ratios (RRs) with 95% confidence intervals (CIs). Continuous data were analysed as the mean difference with inverse variance. We assessed statistical heterogeneity using the I^2^ statistic, interpreting the results as shown in Table 2 [16].

**Table 2:** Assessment of statistical heterogeneity

Value of I^2^	Interpretation
0% to 40%	might not be important
30% to 60%	may represent moderate heterogeneity
50% to 90%	may represent substantial heterogeneity
75% to 100%	considerable heterogeneity

Results

Study Characteristics

**Figure 1:**
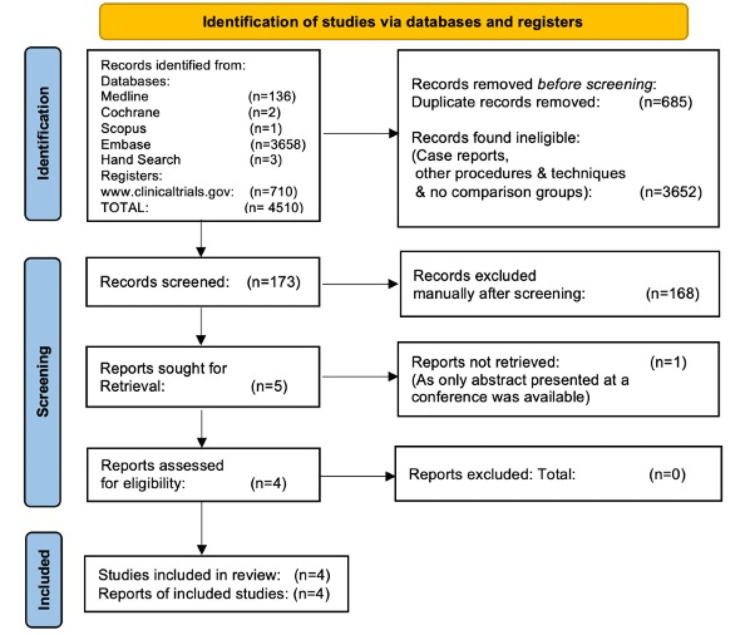
PRISMA flow diagram

We identified 4510 articles with our search criteria and 173 records were screened. Out of these 168 articles were excluded and five articles were assessed for eligibility. One study was further excluded for reasons as only abstract presented at conference was available and outcome details were not available. Finally, four studies [17-20] were included in the meta-analysis. One of the included studies is prospective randomised, one is prospective comparative, one study is bidirectional comparative and the last study is prospective non-randomised in design (Figure 1).

A total of 642 patients were included in these studies including 376 males and 266 females. There were 359 patients in the CT group and 283 patients in the HS group. The baseline characteristics of the included studies are demonstrated in Table 3.

**Table 3:** Study characteristics CT: Conventional technique of appendiceal stump closure, HS: Harmonic scalpel for appendiceal stump closure, NA: Details not available.

SN	Study	Period	Study Design	Total Patients (M/F)	Sample Size		Mean age (yrs.)		Average Follow-up	
					Group 1 CT	Group 2 HS	Group 1 CT	Group 2 HS	Group 1 CT	Group 2 HS
1	Hamdy et al., 2018 [19]	June 2016 to April 2018	Prospective comparative (Loop/Harmonic scalpel)	40 (21 M, 19 F)	20	20	27.85+/-8.96 (18-47)	28.95+/-8.87 (19-48)	NA	NA
2	Bhasin et al., 2019 [20]	NA	Prospective randomized comparative (Extracorporeal knotting/Harmonic scalpel)	80	40	40	27.6 (7-62)	26.9 (8-64)	NA	NA
3	Gupta et al., 2020 [18]	Jan 2015- June 2019	Bidirectional comparative (Endoloop/Harmonic scalpel)	210 (116 M, 94 F)	102	108	29.26+/-11.27 (10-62 yrs)	31.22+/-13.35 (12-65 yrs)	3 months	3 months
4	Pogorelic et al., 2022 [17]	Jan 2019- May 2021	Prospective (Polymeric clip /Harmonic scalpel)	312 (191 M, 121 F) CT-119 M, 78 F; HS-72 M, 43 F	197	115	11 (9,14) Median and IQR	11 (8,15)	1 month	1 month

The population of the included studies are found to be heterogeneous. Out of the four studies, two studies [18,20] have included only adolescent and adult patients, one has included only adult patients [19] while one study [17] has included exclusively the pediatric population. The average age of patients in these studies showed no significant difference between the CT and the HS group.

Results of the Individual Studies

Postoperative complications and outcome assessment reported in each study are summarized in Table 4.

**Table 4:** Outcome chart CT: Conventional technique of appendiceal stump closure, HS: Harmonic scalpel for appendiceal stump closure, SD: Standard deviation, IQR: Interquartile range.

SN	Study	Wound infection		Mean operative time (minutes)		Postoperative ileus		Hospital stay (days)		Postoperative leak		Total complications	
		CT	HS	Group CT	Group 2 HS	Group CT	Group HS	Group CT	Group (HS)	CT	HS	CT	HS
1	Hamdy et al. [19]	2	2	49.95+/-3.63 (45-57)	38.95+/-3.55 (34-46)	NA	NA	3.50+/-1.00 (2-5)	3.45+/-0.83 (2-5)	0	0	2	2
2	Bhasin et al. [20]	3	1	50.8+/-7.17 (30-60)	35.44+/- 6.37 (20-40)	2	3	3.12 (2-5)	2.76 (2-4)	NA	NA	5	4
3	Gupta et al. [18]	4	4	43.34+/-6.7 (29-58)	28.46+/-7.19 (17-48)	8	10	NA	NA	NA	NA	12	14
4	Pogorelic et al. [17]	0	0	30 (22,40) Median IQR Mean 33.51 min, SD 15.67	21 (18,25) Median IQR, Mean 23.01, SD 10.34	3	0	3 (2,4) Median, IQR, Mean 3.56, SD 2.18	2 (2,3) Median, IQR Mean 2.51, SD 1.51	NA	NA	10 (7 abscess)	0

Methodological Quality Assessment

The Downs and Black scoring was done by two authors (SS, DM). The average score of the four studies ranged from 16-20. All the studies are found to have a high risk of bias. The study by Pogorelic et al. [17] has the highest score of 20 and the study by Hamdy et al. [19] has the lowest score of 16. The inter-observer agreement, i.e., Kappa was strong (Kappa=0.87).

Outcome analysis

Meta-Analysis of Outcome - Mean Operative Time

**Figure 2:**
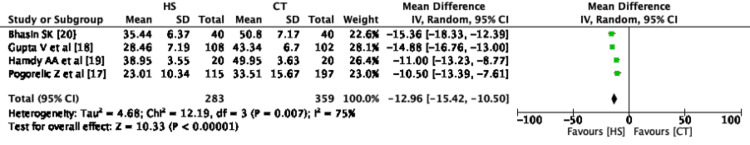
Forest Plot - Mean operative time

All four studies have depicted this outcome. Three studies have depicted the mean operative time in terms of mean with standard deviation while one study [17] has depicted it with median and IQR. In the mail correspondence with the author of the last study 17 has provided us with the desired details. There were 283 events in the HS group and 359 events in the CT group. Pooled data between CT and HS groups showed that the mean operative time is less in the HS group with a pooled mean difference of -12.96 with 95% CI -15.42, -10.50. We observed that there is substantial statistical heterogeneity of (I^2^- 75%) of the included studies (Figure 2).

*Meta-Analysis of Outcome - Postoperative Ileus* 

**Figure 3:**
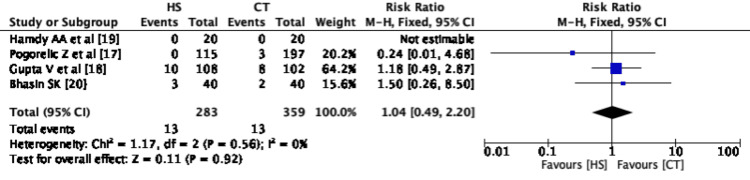
Forest Plot - Postoperative ileus

Out of four studies included in the meta-analysis, three studies have reported this outcome. There were 13 events in the CT group among 283 patients (4.59%) and 13 events in the HS group among 359 patients (3.62%). The pooled risk ratio between HS and CT groups showed no significant difference in postoperative ileus between both the groups (RR 1.04, CI 0.49-2.20) (Figure 3). There is no statistical heterogeneity observed among the included studies (I^2^=0%) (Figure 3).

Meta-Analysis of Outcome - Wound Infection

**Figure 4:**
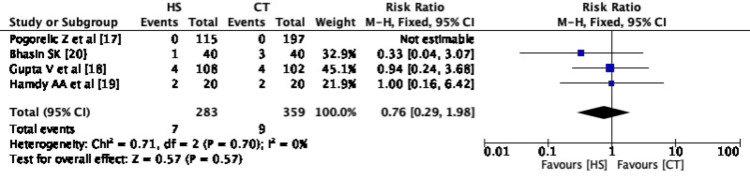
Forest Plot - Wound infection

Out of the four studies three have reported wound infection/port site infection following LA. There were no wound infections observed in the study by Pogorelic et al. [17]. There were nine patients (2.50%) with wound infections in the CT group and seven patients (2.47%) with wound infections in the HS group. The pooled risk ratio between CT and HS groups shows no statistically significant difference between both the groups (Figure 4).

Meta-Analysis of Outcome - Hospital Stay

**Figure 5:**
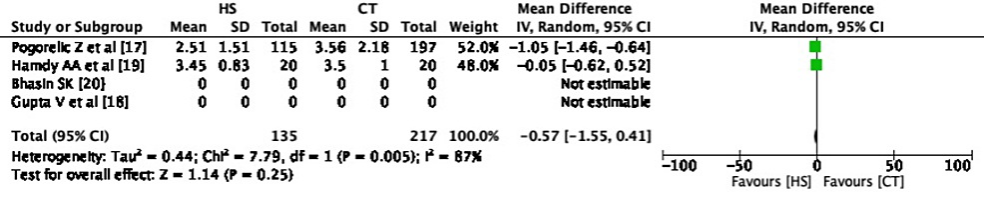
Forest Plot - Hospital stay

All four studies except that by Gupta et al. [18] have mentioned the duration of hospital stay following LA. Hamdy et al. [19] reported hospital stay as mean and range so his study was excluded from the pooled analysis as standard deviation was not provided. Though Gupta et al. have not mentioned the postoperative stay, telephonic conversion with the corresponding author confirmed that it was statistically insignificant in both the groups. Pooled analysis of the two remaining studies showed no significant difference between hospital stay in both the groups with a pooled mean difference of -0.57 and CI -1.55, 0.41 (Figure 5).

Meta-Analysis of Outcome - Total Complications

**Figure 6:**
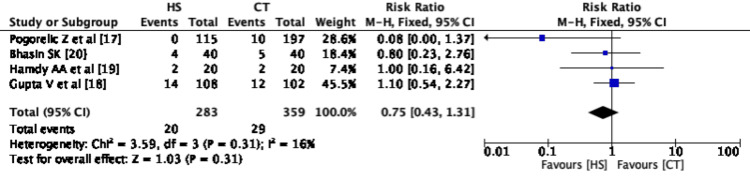
Forest plot - Total complications

One study has summarized the outcome measure of total complications and in the rest of the studies, it was summarized by the reviewing authors. Total complications in the CT group were 29 (8.07%) and 20 (7.06%) in the HS group. Pooled risk ratio between both the groups for total complications is non-significant with a RR of 0.75 with 95% CI 0.43-1.31 (Figure 6).

Discussion

The present meta-analysis evaluates the performance of HS used for sealing the base of the appendix in LA.

Summary of Results

A pooled analysis of the outcome measure of total complications shows that using HS for closure of appendiceal stump does not result in an increased incidence of complications as compared to the conventional technology of appendiceal stump closure. The pooled analysis of the outcome measure of mean operative time has demonstrated statistically significant reduced operative time in the group where HS has been used for managing the appendiceal stump as compared to CT (pooled mean difference of -12.96 with 95% CI -15.42, -10.50). A significantly shorter hospital stay has been reported by Pogorelic et al. [17]; however, the pooled analysis shows that there is no significant difference between both the groups for this outcome.

Minimal invasive techniques require special instrumentation to ensure the safety and efficacy of surgical procedures. The HS is one of such instruments which is regularly used in minimally invasive surgery, especially laparoscopy. This instrument utilizes ultrasonic energy by converting it to mechanical energy. The HS assists in tissue coagulation and cutting through the vibration of the active blade. The high-frequency vibration induces stress and friction in the tissue, which leads to intense heat generation and protein denaturation. The working principle of HS includes a combination of applying pressure and then sealing with a denatured protein coagulum while applying ultrasonic vibration to denature hydrogen bonds to perform vessel coagulation [21,22]. Ethicon first introduced HARMONIC ultrasonic technology and its precise dissection capability to the world in 1998 with the release of the HARMONIC scalpel shears [22]. In LA the HS is a handy instrument for the dissection and division of the mesoappendix. A study by Qaiser et al. [23] has shown that HS was better than suture ligation for managing the mesoappendix in LA in terms of mean operative time and control of intraoperative bleeding. Its main advantages include precise dissection, reliable hemostasis, less lateral thermal spread and charring.

Primarily used for vessel sealing and dissection, the use of HS has been extended to the sealing of luminal structures other than blood vessels such as cystic duct and appendix. A meta-analysis comparing clip ligation and division of cystic duct with HS concluded that HS contributed to reduced operative time as well as hospital stay during laparoscopic cholecystectomy as compared with conventional clips [9]. The use of HS for cystic duct division was also comparable to clips concerning conversion to open cholecystectomy, perforation of gallbladder, bile leakage and overall morbidity [9].

Apart from comparative studies used for this meta-analysis, there is a large case series of 63 patients by Raza et al. [24] where appendiceal stump closure was performed with HS. None of the patients experienced any complications in this series and the mean operative time for LA was 31.4 minutes.

The use of HS for stump closure has several advantages over the other methods of appendiceal stump sealing. The division of the mesoappendix, as well as the appendix stump sealing and division, can be performed with a single shear of HS. So, HS obviates the need for a change of the hand instruments like needle holder, knot pusher, endoclips, and GIA stapler for stump closure. These factors contribute to the reduction in the mean operative time. The shear of HS can be introduced through a standard 5 mm port in contrast to the need for a 10-12 mm port for endo GIA application. It also avoids foreign body reactions and reduces the risk of postoperative adhesion formation [18].

The major drawback of HS is the cost of disposable hand instruments. As per the manufacturer, the HS shears are meant for one-time use only. A new shear is supposed to be used for each surgery; however, a recent RCT has supported the reuse of the HS shears in settings of economic constraints [25]. The adverse economic impact of the high-cost hand shears can be offset to an extent by the reduced operative time which translates into effective utilisation of resources in terms of less consumption of anaesthetic drugs and an increase in operation theatre turnover. The precise bloodless dissection offered by the HS shear can also contribute to faster recovery of the patients and early return to productive work. There should be well-planned RCTs to find out the economic aspects of HS versus conventional techniques in LA concerning operative time, cost-benefit and resource utilisation.

Limitation of the Study

A limited number of comparative studies are available for this meta-analysis. In addition, substantial heterogeneity was observed among the studies included for the outcome analysis of mean operative time. Subgroup analysis among the pediatric and adult population could not be performed due to availability of a single study in each population group in our review.

## Conclusions

Appendiceal stump closure by harmonic scalpel is comparable with the conventional techniques in LA in terms of hospital stay, wound infection, and postoperative paralytic ileus, as well as the incidence of total complication. The use of HS for closure of appendicular stump during LA accounts for a reduction in the mean operative time. We propose undertaking further RCTs with larger sample sizes to address the economic constraints associated with advanced energy devices for widespread acceptance of sutureless appendicectomy.
